# Development of a Lateral Flow Strip-Based Recombinase-Aided Amplification for Active *Chlamydia psittaci* Infection

**DOI:** 10.3389/fmicb.2022.928025

**Published:** 2022-06-13

**Authors:** Jun Jiao, Yong Qi, Peisheng He, Weiqiang Wan, Xuan OuYang, Yonghui Yu, Bohai Wen, Xiaolu Xiong

**Affiliations:** ^1^State Key Laboratory of Pathogen and Biosecurity, Beijing Institute of Microbiology and Epidemiology, Beijing, China; ^2^Huadong Research Institute for Medicine and Biotechniques, Nanjing, China

**Keywords:** *Chlamydia psittaci*, detection, recombinase-aided amplification, lateral flow strip, infection

## Abstract

*Chlamydia psittaci* is the causative agent of psittacosis, a worldwide zoonotic disease. A rapid, specific, and sensitive diagnostic assay would be benefit for *C. psittaci* infection control. In this study, an assay combining recombinase-aided amplification and a lateral flow strip (RAA-LF) for the detection of active *C. psittaci* infection was developed. The RAA-LF assay targeted the *CPSIT_RS02830* gene of *C. psittaci* and could be accomplished in 15 min at a single temperature (39°C). The analytical sensitivity of the assay was as low as 1 × 10^0^ copies/μl and no cross-reaction with some other intracellular pathogens was observed. Moreover, all feces samples from mice infected with *C. psittaci* at day-1 post-infection were positive in the RAA-LF assay. In conclusion, the RAA-LF assay provides a convenient, rapid, specific and sensitive method for detection of active *C. psittaci* infection and it is also suitable for *C. psittaci* detection in field.

## Introduction

Zoonoses are major and global challenges for public health (Xiaoping and Lynn, 2021)*. Chlamydia psittaci*, an obligate intracellular Gram-negative bacterium with a unique developmental cycle, is the causative agent of psittacosis, a worldwide zoonotic disease. More than 467 bird species and many nonavian domestic animals, including swine, dogs, horses, cattle, sheep, goats, and cats, as well as rodents are susceptible to *C. psittaci* (Pantchev et al., 2010; Lenzko et al., 2011; Knittler and Sachse, 2015; Gu et al., 2020). *C. psittaci* infection in humans could occasionally occur after inhalation of *C. psittaci-*contaminated aerosols from infected birds/nonavian animals (Wolff et al., 2018). Due to low awareness of the disease and atypical clinical presentation in majority of the cases, psittacosis in humans is underestimated (Gu et al., 2020).

The burden of *C. psittaci* infection is also likely underestimated due to diagnostic challenges: the bacterium requires intracellular growth and is a zoonotic human pathogen responsible for atypical pneumonias that may be caused by various pathogens such as *Mycoplasma pneumonia*, *Legionella pneumophila and Coxiella burnetii* (Cunha, 2006; Jiao et al., 2014). To date, several assays have been developed for *C. psittaci* detection. Conventional methods including pathogen isolation, indirect immunofluorescence, enzyme linked immunosorbent assay (ELISA), complement fixation, outer membrane protein A (ompA) sequencing and restriction fragment length polymorphism analysis have been used (Souriau and Rodolakis, 1986; Sanderson et al., 1994; Mitchell et al., 2009a,b; Opota et al., 2015), but these methods are laborious, low sensitive, time-consuming and also require a trained personnel. The PCR-based diagnostic methods such as conventional PCR, multi-PCR, and real-time PCR have been developed to detect *C. psittaci* (Hewinson et al., 1997; Messmer et al., 1997; Wolff et al., 2018). Although these PCR-based methods are highly sensitive, the requirement of expensive instruments and skilled operators make the use of these methods difficult in resource-limited settings.

Recombinase-aided amplification (RAA) assay is a new isothermal amplification technology for pathogens detection with the advantages of rapidity, simplicity, and low cost (Chen et al., 2018; Duan et al., 2018; Yan et al., 2018; Wang et al., 2020; Ruichen Lv et al., 2022). This amplification process only requires approximately 15–30 min at 39°C and has been successfully applied for detection of African Swine Fever Virus (Fan et al., 2020), *Klebsiella pneumoniae* (Hou et al., 2021), human norovirus GII.4 (Qin et al., 2021), duck circovirus (Li et al., 2021), *Trypanosoma evansi* (Li et al., 2020), etc. Additionally, the result of RAA assay can be observed on the lateral flow strip (LF) by naked eyes, providing a convenient and rapid diagnostic assay for the detection of the target pathogens, and thus it is suitable for clinical application and/or testing in field.

For *Chlamydia* spp. detection, urethral swabs, choanal cleft swabs, pharyngeal swab, and cloacal swabs from animals or human are often used for DNA extraction and testing (Holland et al., 1990; Dumke et al., 2015; Mina et al., 2019; Feodorova et al., 2022). One route of transmission of *C. psittaci* is a inhalation of aerosolized bird feces from the environment (Szeredi et al., 2005), and fresh feces samples from animals are also used for detection of *C. psittaci* (Takashima et al., 1996; Sachse et al., 2012; McGovern et al., 2021). Another advantage for the choice of fresh feces as a key specimen for detection of *C. psittaci* maybe that a feces collection is more convenient than a blood collection: rapid, harmless, and suitable for detection in filed. In the present study, a convenient and rapid recombinase-aided amplification and a lateral flow strip (RAA-LF) assay was developed for the detection of active *C. psittaci* infection, and its sensitivity and specificity were investigated. In addition, the effectiveness of the RAA-LF assay was evaluated with feces samples from mice experimentally infected with *C. psittaci*.

## Materials and Methods

### Bacteria and DNA Samples

*Chlamydia psittaci* strain 6BC was cultured in buffalo green monkey kidney (BGMK) cells, and then the whole DNA were extracted from the cells infected with the strain using a QIAamp Blood and Tissue Mini DNA kit (Qiagen, Hilden, Germany) according to the manufacturer’s instructions. The DNA sample was eluted in 100 μl of nuclease-free water and determined by quantitative PCR (qPCR) as described previously (Menard et al., 2006).

DNA samples of *Anaplasma phagocytophilum*, *Rickettsia heilongjiangensis, Rickettsia rickettsii*, *Rickettsia sibirica, Rickettsia canada*, *Rickettsia australis*, *Rickettsia typhi*, *Rickettsia prowazekii*, *Ehrlichia chaffeensis*, *C. burnetii* (strain Henzerling and QIYI), *L. pneumophila*, *Listeria monocytogenes*, *Salmonella typhimurium*, *Staphylococcus aureus*, *Vibrio cholera*, *Bartonella henselae*, *Bartonella quintana*, *Streptococcus suis*, *Shigella sonnei* and *Chlamydia trachomatis* were preserved in our laboratory.

Deoxyribonucleic acid samples extracted from fresh feces of *C. psittaci*-infected mice were also provided. Briefly, specific-pathogen free (SPF) BALB/c mice (female, 6–8 weeks old) were divided into two groups with four mice per group and each mouse was infected with 1 × 10^5^ *C. psittaci* organisms suspended in 50 μl phosphate buffer saline (PBS) *via* intranasal administration. All individuals in Group 2 were intraperitoneally administered with tetracycline (40 mg per kg) at day 7 post-infection. Fresh feces samples from mice in both groups were collected at days 1, 3, 7, 10, 14, 17 and/or 20 post-infection, and DNA was extracted from each feces sample using a QIAamp Blood and Tissue Mini DNA kit (Qiagen, Hilden, Germany). The purified DNA was eluted in 100 μl of nuclease-free water.

### Primers and Probes

The nucleotide sequences of the *CPSIT_RS02830* gene of several *C. psittaci* strains were aligned and their conserved regions were selected to design the primers and probes. The designed primers and probe were blasted against nucleotide database (GenBank) in the National Center for Biotechnology Information^1^ to confirm their specificity. The primers and probe were synthesized by GenScript Biotech (Nanjing, China).

### RAA Assay and Lateral Flow Reading

The RAA assays were performed in 50-μl reaction volumes using a commercial RAA kit (ZC Biosicence, Hangzhou, China). The reaction mixture contained 4 μl of extracted DNA template, 2.5 μl of distilled water, 37.9 μl of reaction buffer A, 2 μl of primer F (2.5 μm), 2 μl of primer R (2.5 μm), and 0.6 μl of probe (2.5 μm). The reaction mixture was added to a tube containing the RAA enzyme mix, and then 1 μl of reaction buffer B was added to initiate the reaction. The tubes were transferred to a metal bath (Coyote, Beijing, China) at 39°C for 15 min. A negative control (blank) was included in each run. After the reaction, 10 μl of the amplified product was diluted in 90 μl PBS, and then 50 μl of the diluted sample was transferred to the sample pad of a Milenia GenLine HybriDetect strip (Milenia Biotec, Gieben, Germany) and incubated 3 min at room temperature. Results were judged visually by naked eyes, and a positive result was determined when both the low line of test (T) and the upper line of control (C) developed. Only C line occurrence indicated a negative result, and if the C line did not develop, the strip should be replaced. The assays were performed in triplicate.

### Evaluation of the RAA-LF Conditions

The optimal RAA-LF reaction time and temperature were determined by examining different time settings ranging from 5 to 30 min and various temperature settings ranging from 37 to 42°C. For evaluation of the specificity, 1 × 10^5^ copies/μl of *C. psittaci* DNA and at least 1 × 10^4^ copies/μl of DNA templates from other bacteria were utilized in a 50 μl RAA reaction mixture. For evaluation of the sensitivity, the RAA-LF reaction was evaluated using 10-fold serial dilutions of *C. psittaci* DNA, ranging from 1 × 10^0^ copy to 1 × 10^5^ copies/μl.

### Evaluation of the RAA-LF Assay Using Experimental Samples

To evaluate the performance of the RAA-LF assays for the detection of active *C. psittaci* infection, specific-pathogen free (SPF) BALB/c mice (*n* = 8, female, 6–8 weeks old) were divided into two groups with four mice per group, and each mouse was infected with 1 × 10^5^ *C. psittaci* strain 6BC suspended in 50 μl phosphate buffer saline (PBS) *via* intranasal administration. Fresh feces samples from mice in both the groups were collected at days 1, 3, 7, 10, 14, 17, and/or 20 post-infection and DNA extracted from fresh feces obtained from experimentally infected mice was subjected to RAA-LF or qPCR. Fresh feces collected from naïve mice (*n* = 4, female, 6–8 weeks old) were used as negative control. RAA-LF amplification was performed as mentioned above, and qPCR was performed as previously described (Menard et al., 2006).

## Results

### Designing of Primers and Probes for RAA

**Figure 1:**
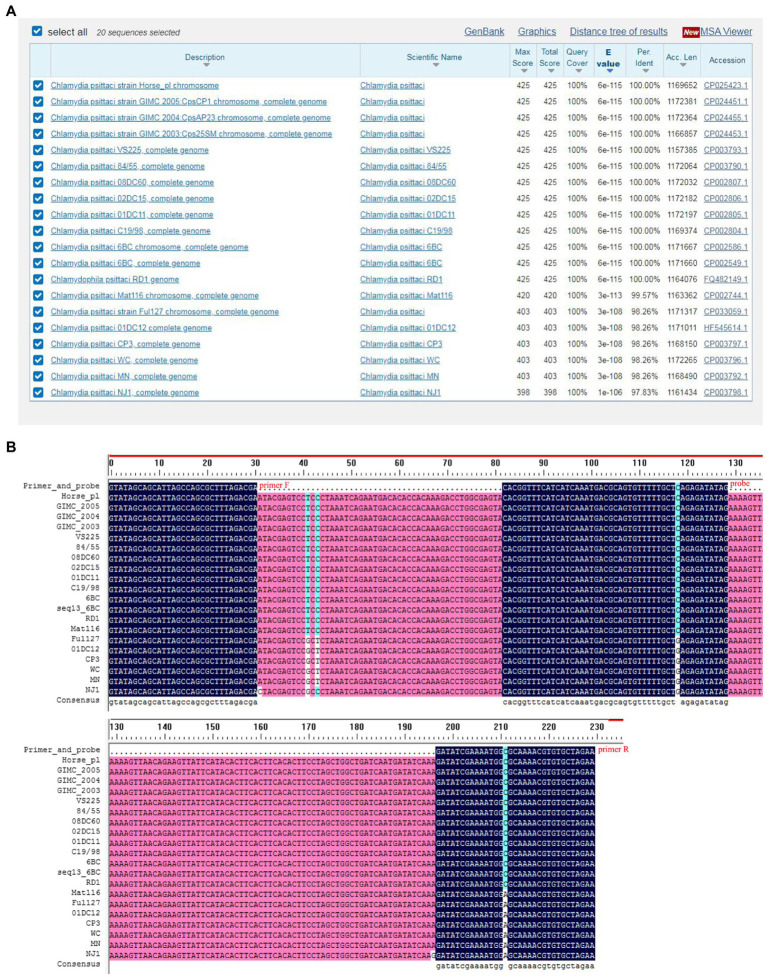
Alignment of the conservation of the *CPSIT_RS02830* gene. The conservative sequence of the *CPSIT_RS02830* gene in *Chlamydia psittaci* was blasted against the National Center for Biotechnology Information nucleotide database **(A)**. The target sequences of the *CPSIT_RS02830* gene was aligned between the forward and reverse primers among various *C. psittaci* strains **(B)**.

Screening of specific primer pairs and probes is the critical step for RAA assay. In the present study, the conservation of *CPSIT_RS02830* gene sequence among 20 *C. psittaci* strains was evaluated by alignment (Figure 1A). As a result, the sequence between primers F and R was aligned with the corresponding sequence in all 20 *C. psittaci* strains but without that of another *Chlamydia* species such as *C. trachomatis* or *C. abortus*, and only one base in the probe mismatched with that of several strains, indicating the target sequence was conserved and suitable for the detection for *C. psittaci* (Figure 1B and Table 1).

**Table 1:** Sequences of the primers and probes used for the RAA amplification.

Primer/probe	Sequence (5′–3′)	Source of the primer/probe	Sequence of product
F	GTATAGCAGCATTAGCCAGCGCTTTAGACGA	This study	GTATAGCAGCATTAGCCAGCGCTTTAGACGAATACGAGTCCTCCCTAAATCAGAATGACACACCACAAAGACCTGGCGAGTACACGGTTTCATCATCAAATGACGCAGTGTTTTTGCTCAGAGATATAGAAAAGTTAACAGAAGTTATTCATACACTTCACTTCACACTTCCTAGCTGGCTGATCAATGATATCAAAGATATCGAAAATGGCGCAAAACGTGTGCTAGAA
R	Biotin-TTCTAGCACACGTTTTGCGCCATTTTCGATATC	This study	
Probe	Fam-CACGGTTTCATCATCAAATGACGCAGTGTT[THF]TTTGCTCAGAGATATAG-Phosphate	This study	

### Evaluation of the RAA-LF Reaction Time and Temperature

**Figure 2:**
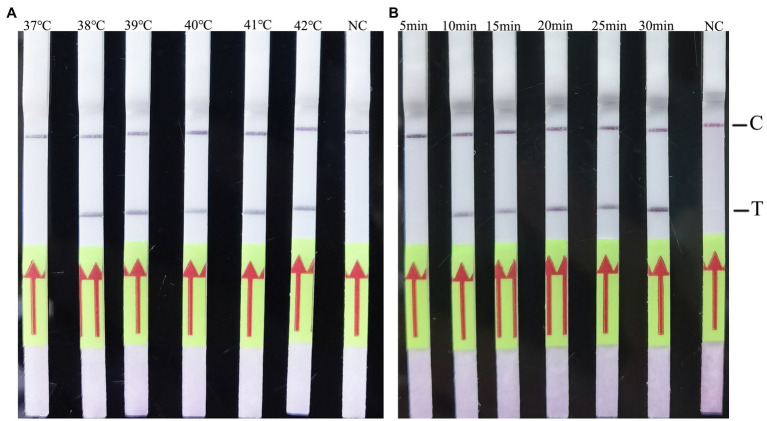
Evaluation of the amplification temperatures and amplification times on the RAA-LF assay. 1 × 10^5^ copies/μl of *C. psittaci* DNA was utilized in a 50 μl RAA reaction mixture and deionized water was provide as negative control. A positive result was determined when both the low line of test (T) and the upper line of control (C) developed. Only C line occurrence indicated a negative result. The RAA-LF worked well in a wide range of amplification temperature from 37 to 42°C **(A)**. After 5–30 min of isothermal amplification reaction, the positive reaction was visible on the test strip **(B)**.

The RAA-LF reaction temperature was tested at a wide range of temperatures from 37 to 42°C. The results showed that the reaction performs well at 38–42°C after 3 min incubation on the lateral flow strip (Figure 2A). Then the RAA-LF amplification was achieved at times ranging from 5 to 30 min at 39°C. The results showed that a bright T line could be detected when the amplification time was 10 min, and it became brighter as the amplified time increased to 30 min (Figure 2B).

### Analytical Specificity and Sensitivity of the RAA-LF Assay

**Figure 3:**
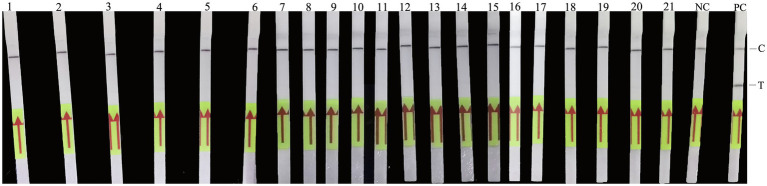
Analytical specificity of the RAA-LF assay. Line 1: *Anaplasma phagocytophilum*; Line 2: *Rickettsia heilongjiangensis*; Line 3: *R. rickettsia*; Line 4: *R. sibirica*; Line 5: *R. Canada*; Line 6: *R. australis*; Line 7: *R. typhi*; Line 8: *R. prowazekii*; Line 9: *Ehrlichia chaffeensis*; Line 10: *Coxiella burnetii* (Henzerling); Line 11: *C. burnetii* (QIYI); Line 12: *Legionella pneumophila*; Line 13: *Listeria monocytogenes*; Line 14: *Salmonella typhimurium*; Line 15: *Staphylococcus aureus*; Line 16: *Vibrio cholera*; Line 17: *Bartonella henselae*; Line 18: *Bartonella quintana*; Line 19: *Streptococcus suis*; Line 20: *Shigella sonnei*; Line 21: *Chlamydia trachomatis*; NC: negative control; PC: *C. psittaci*. A positive result was determined when both the low line of test (T) and the upper line of control (C) developed. Only C line occurrence indicated a negative result.

To evaluate the specificity of the developed RAA-LF assay, DNA samples belong to *Rickettsia*, *Legionella*, and several other pathogenic bacteria were assessed. Our results showed that a clearly T line could be observed by the naked eyes only for DNA sample of *C. psittaci*, and there was no cross-reactivity with DNA samples of *A. phagocytophilum*, *R. heilongjiangensis*, *R. rickettsia*, *R. sibirica, R. Canada*, *R. australis*, *R. typhi*, *R. prowazekii*, *E. chaffeensis*, *C. burnetii* (Henzerling and QIYI), *L. pneumophila*, *L. monocytogenes*, *S. typhimurium*, *S. aureus*, *V. cholera*, *B. henselae*, *B. quintana*, *S. suis*, *S. sonnei* and *C. trachomatis* (Figure 3).

**Figure 4:**
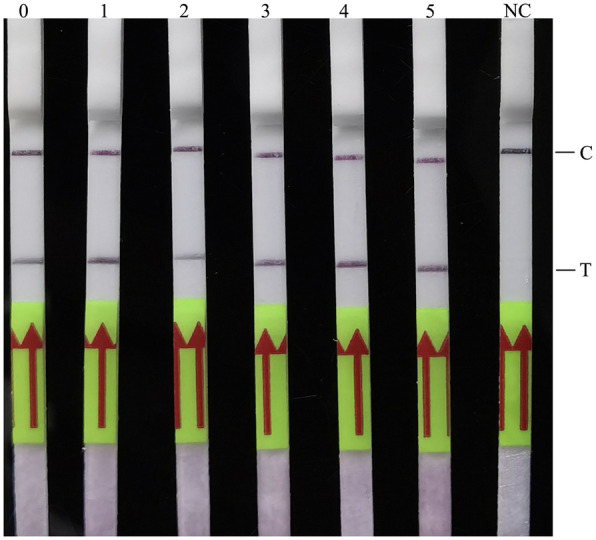
Analytical sensitivity of the RAA-LF assay. Ten-fold serial dilutions of *C. psittaci* genomic DNA were evaluated by the RAA-LF assay, and deionized water was provided as negative control. A positive result was determined when both the low line of test (T) and the upper line of control (C) developed. Only C line occurrence indicated a negative result. Lines 0–5: 1 × 10^0^ copy to 1 × 10^5^ copies/μl. NC: negative control.

To evaluate the sensitivity of the developed RAA-LF assay, 10-fold serial dilutions of *C. psittaci* DNA, ranging from 1 × 10^0^ copy to 1 × 10^5^ copies/μl were used. Results showed that the developed RAA-LF assay was highly sensitive with a detection limit as low as 1 × 10^0^ copies/μl of *C. psittaci* (Figure 4).

### The RAA-LF Assay Can Detect Active *Chlamydia psittaci* Infection in an Experimental Mouse Model

In the experiment, BALB/c mice infected with *C. psittaci* were divided into two groups. Mice in group 1 were left untreated, while mice in group 2 were treated with tetracycline at day-7 post-infection. Fresh feces samples from mice of both the groups were collected at days 1, 3, 7, 10, 14, 17, and/or 20 post-infection, and DNA extracted from feces samples were detected by the developed RAA-LF assay.

**Figure 5:**
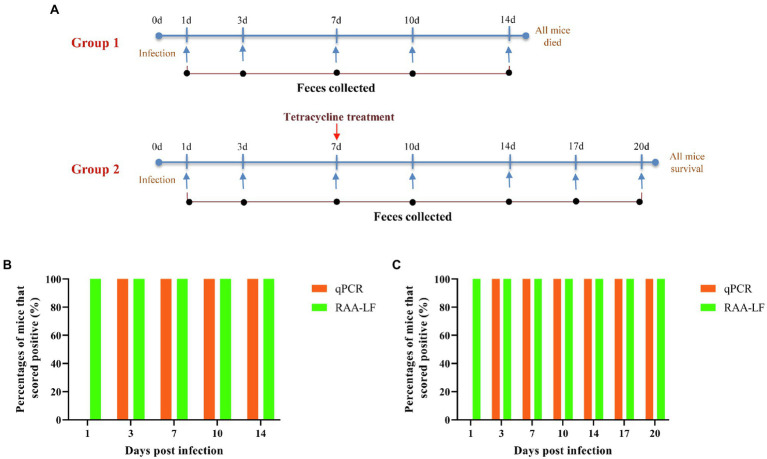
Results of the evaluation of qPCR and the RAA-LF assay for experimentally infected samples. BALB/c mice infected with *Chlamydia psittaci* were divided into two groups of four individuals. Group 1 was left untreated, while Group 2 was treated with tetracycline at 7 days post-infection. All mice in group 1 died at day 15 post-infection and all mice in group 2 were survival **(A)**. Fresh feces samples from group 1 **(B)** and group 2 **(C)** were collected at days 1, 3, 7, 10, 14, 17 and/or 20 post-infection, and the extracted DNA from fresh feces samples were evaluated by qPCR and the RAA-LF assay. The results were displayed as the percentages of mice that scored positive (orange) or negative (green).

As shown in Figure 5, the two techniques did not yield identical results for all of the feces samples. All of the feces samples were detected positive in the RAA-LF assay. However, none of the feces samples from the infected mice at day 1 post-infection was detected positive in *C. psittaci*-specific qPCR (Figure 5; Supplementary Table S1 and Supplementary Figure S1), suggesting that the RAA-LF assay is more sensitive for detection of an early *C. psittaci* infection compared with qPCR. All of the feces samples in group 1, which contained around 10^3^–10^4^ copies/μl of genomic DNA as determined in qPCR at days 3, 7, 10, and 14 post-infection (Supplementary Table S1), were also detected positive in the RAA-LF assay. Unfortunately, all mice in group 1 died at day 15 post-infection.

In group 2, all mice were survival and their feces samples were detected with decreasing levels of *C. psittaci* from day 7 post-infection (Supplementary Table S1). All of the feces samples from day 3 post-infection were detected positive in *C. psittaci-*specific qPCR and RAA-LF assay, indicating that the RAA-LF is a suitable assay for visual detection of active *C. psittaci* infection under the experimental condition. The feces samples from naïve mice were also detected and there was no positive result in the RAA-LF assay (Supplementary Figure S1).

## Discussion

*Chlamydia psittaci* is a zoonotic agent of systemic wasting disease in birds and atypical pneumonia in mammalians including humans, constituting a public health risk (Knittler and Sachse, 2015). Human infections caused by *C. psittaci* are underestimated, which is mainly due to the difficulty in diagnosing this disease. A convenient, rapid, specific and sensitive method for the detection of *C. psittaci* infection is needed in order to achieve an effective psittacosis control. In the present study, we have developed a convenient and rapid RAA-LF assay for detection of active *C. psittaci* infection.

Evaluation of specificity of a detection assay using unrelated bacteria DNA is of equal importance as using related bacteria DNA (Sails et al., 2003; Gumaa et al., 2019). The RAA-LF assay was evaluated using the whole DNA samples of *C. psittaci* or another intracellular pathogenic bacteria which are typically neglected in causing fever of unknown origin (*Rickettsia* spp. or *Legionella* spp.), or another unrelated pathogenic bacterium (*S. typhimurium*, *S. aureus* etc.). Specially, DNA of *V. cholera*, a pathogenic bacterium which is commonly transmitted by drinking water contaminated by the patients’ feces, was also used as an unrelated sample in the present study. As a result, only DNA samples of *C. psittaci* were detected positive in the RAA-LF assay, suggesting that this RAA-LF assay performs a good specificity. Further studies should focus on verifying potential cross-reactivity with DNA from other species belong to *chlamydia* spp., like *C. abortus*.

The developed RAA-LF assay is also highly sensitive and the detected limitation is as low as 1 × 10^0^ copies/μl of *C. psittaci* genomic DNA, which is almost 20-fold more sensitive compared with that of the recombinant polymerase amplification (RPA/RAA) assay developed by Pang et al. (2021), making it useful for assessing the samples from the patients in the early acute phase of *C. psittaci* infection. Actually, three forward primers, three reverse primers, and two probes targeting the conservation of *CPSIT_RS02830* gene sequence have been designed and combined to generate 18 different combinations, and then these combinations have been screened for the RAA-LF assay (data not shown). The best combination is the one that descried in Table 1. This may be one explanation of the high sensitivity of the developed RAA-LF assay. In addition, compared with conventional PCR and real-time PCR requiring more than 1 h to complete in a well-equipped laboratory (Herring, 1991; Rasmussen et al., 1992; Sykes et al., 2001; Angen et al., 2021), the time required of the RAA-LF assay is also shorter and could be completed in less than 20 min. Moreover, the RAA-LF assay works well in a wide range of temperature (38–42°C) which is lower than the ones required in loop-mediated isothermal amplification (LAMP) assay (Njiru, 2012). Therefore, this RAA-LF assay does not require any expensive instrument and can be performed in minimally equipped laboratories and even in field.

The RAA-LF assay was evaluated for its potential to detect active *C. psittaci* infection at various time points in an experimental mouse model. The results showed that the RAA-LF assay exhibits a potential suitable for direct detection of early *C. psittaci* infection. Feces, as the primary test sample, has potential for PCR inhibition and ultimately reduced analytical and diagnostic sensitivity (Acharya et al., 2017), what’s important is that the complex compositions of feces seems to have not much effects on the test results in the present study. The *C. psittaci* could be detected from fresh feces samples of infected mice at day 1 post-infection, also indicating the available for assessing patient samples in the acute phase. Moreover, an important feature of the method is the results can also be visualized with naked eyes. Therefore, the RAA-LF assay is more suitable for usage under field conditions.

However, there are several limitations in evaluation of the developed RAA-LF assay. Firstly, for lacking of genomic DNA, no various strains of *C. psittaci* or other *Chlamydia* spp. strains except *C. trachomatis* were tested in the present study. Secondly, no clinical samples of patients with psittacosis were used to verify the developed RAA-LF assay. And the addressing of these limitations will contribute to the further preciseness of this study.

## Conclusion

A method for the detection of active *C. psittaci* infection based on a RAA-LF was developed in the present study. The RAA-LF assay can be performed within 20 min using a portable device and positively detect as low as 1 × 10^0^ copies/μl of DNA. In addition, the assay results can be visualized with naked eyes. Therefore, it is a convenient, rapid, sensitive and specific assay for detection of active *C. psittaci* infection, and it is suitable for use in minimally equipped laboratories even field settings.

## Data Availability Statement

The original contributions presented in the study are included in the article/Supplementary Material, further inquiries can be directed to the corresponding author.

## Ethics Statement

The animal study was reviewed and approved by the Institutional Animal Care and Use Committee (IACUC) of the Academy of Military Medical Science.

## Author Contributions

This study was conceived and designed by JJ and XX. Samples collection and laboratory work were performed by PH, XO, and YY. Experimental data analysis was performed by YQ and WW. The manuscript was drafted by JJ and XX, and edited by BW. All authors contributed to the article and approved the submitted version.

## Funding

This work was supported by the National Natural Science Foundation of China [grant numbers 32000139 and 31970178] and the National Key Research and Development Program of China [grant numbers 2019YFC1200500 and 2019YFC1200602].

## Conflict of Interest

The authors declare that the research was conducted in the absence of any commercial or financial relationships that could be construed as a potential conflict of interest.

## Publisher’s Note

All claims expressed in this article are solely those of the authors and do not necessarily represent those of their affiliated organizations, or those of the publisher, the editors and the reviewers. Any product that may be evaluated in this article, or claim that may be made by its manufacturer, is not guaranteed or endorsed by the publisher.

## Footnotes

^1^ https://www.ncbi.nlm.nih.gov/

## Supplementary Material

Supplementary Figure S1 Evaluation of the RAA-LF assay for detection of active *Chlamydia psittaci* infection in mice. Fresh feces samples from group 1 **(A)**, group 2 **(B)** and naïve mice **(C)** were tested by the developed RAA-LF assay. 1, 3 7, 10, 14, 17, 20: days at post-infection. N: negative control. N1–N4: naïve mice. A positive result was determined when both the low line of test (T) and the upper line of control (C) developed. Only C line occurrence indicated a negative result.
