# Supplementary figures and images for: Development of a Lateral Flow Strip-Based Recombinase-Aided Amplification for Active Chlamydia psittaci Infection
Source: Front Microbiol. 2022 Jun 13;13:928025. doi: 10.3389/fmicb.2022.928025 (PMC9234530; doi:10.3389/fmicb.2022.928025)

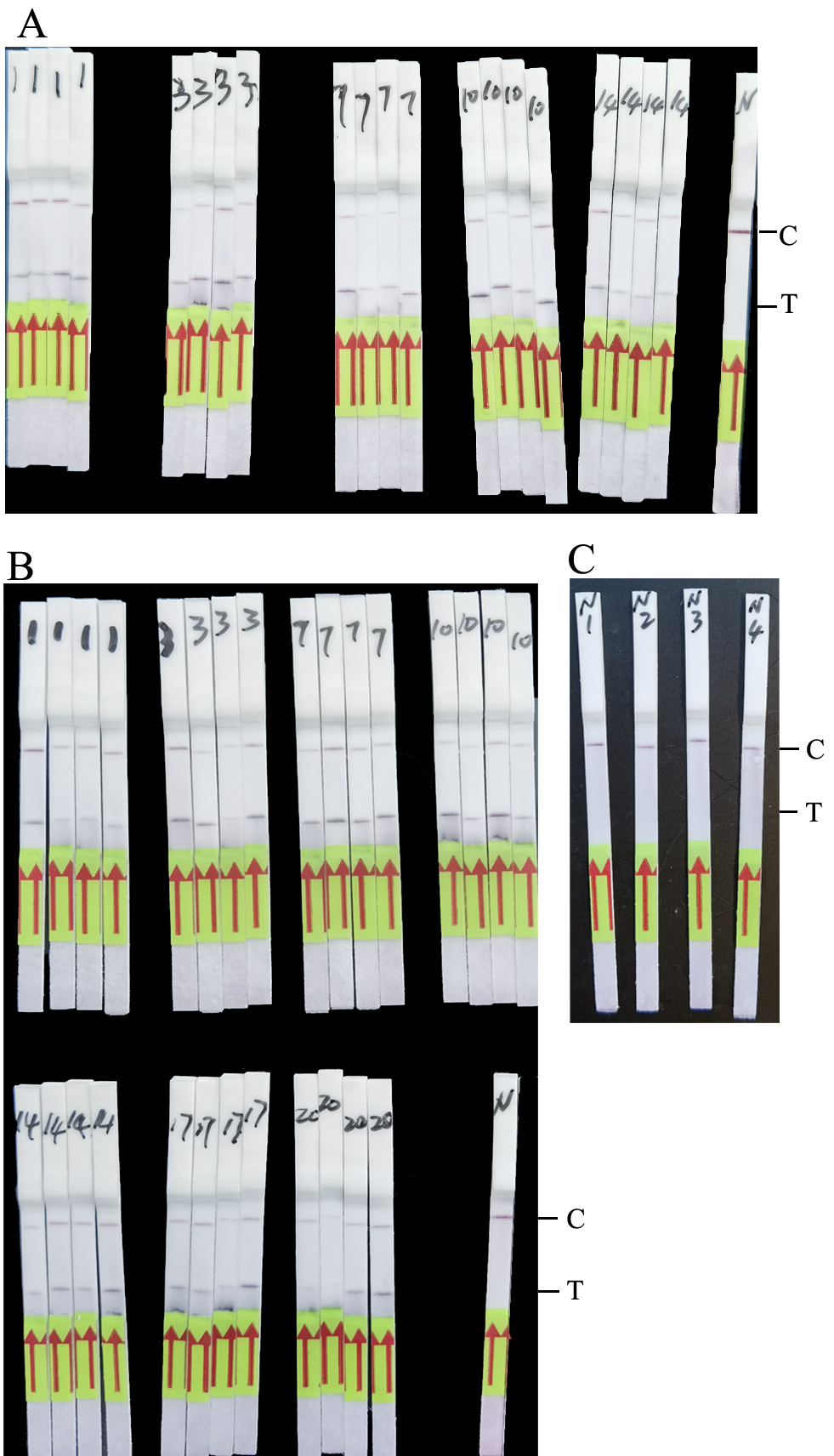

Supplement: Supplementary Figure S1 — Evaluation of the RAA-LF assay for detection of active Chlamydia psittaci infection in mice. Fresh feces samples from group 1 (A), group 2 (B) and naïve mice (C) were tested by the developed RAA-LF assay. 1, 3 7, 10, 14, 17, 20: days at post-infection. N: negative control. N1–N4: naïve mice. A positive result was determined when both the low line of test (T) and the upper line of control (C) developed. Only C line occurrence indicated a negative result. [file Image_1.TIF]
