# Supplementary material for: Development of a Lateral Flow Strip-Based Recombinase-Aided Amplification for Active Chlamydia psittaci Infection
Source: Front Microbiol. 2022 Jun 13;13:928025. doi: 10.3389/fmicb.2022.928025 (PMC9234530; doi:10.3389/fmicb.2022.928025)

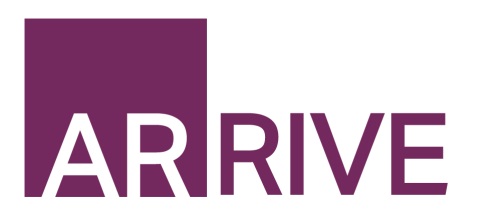


The ARRIVE Guidelines Checklist

Animal Research: Reporting In Vivo Experiments

Carol Kilkenny^1^, William J Browne^2^, Innes C Cuthill^3^, Michael Emerson^4^ and Douglas G Altman^5^

*^1^The National Centre for the Replacement, Refinement and Reduction of Animals in Research, London, UK, ^2^School of Veterinary Science, University of Bristol, Bristol, UK, ^3^School of Biological Sciences, University of Bristol, Bristol, UK, ^4^National Heart and Lung Institute, Imperial College London, UK, ^5^Centre for Statistics in Medicine, University of Oxford, Oxford, UK.*

|  | | ITEM | RECOMMENDATION | Section/ Paragraph |
| --- | --- | --- | --- | --- |
| 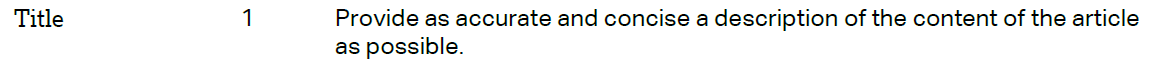 | | | Page 1 |  |
| 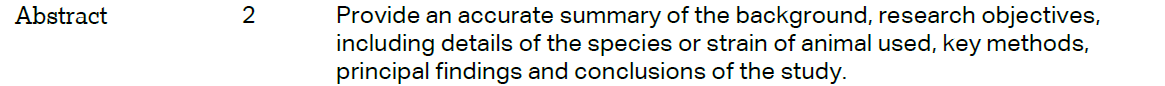 | | | Page 2 |  |
| INTRODUCTION | | |  |  |
| 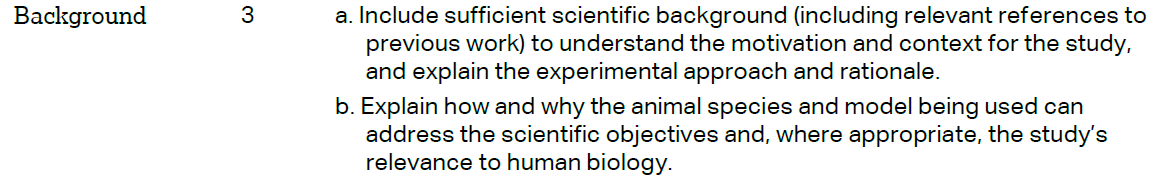 | | | Page 3-4 |  |
| 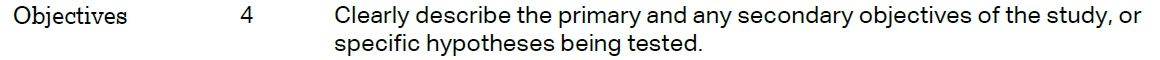 | | | Page 4 |  |
| METHODS | | |  |  |
| 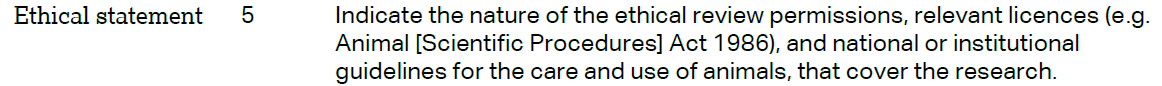 | | | Page 10, |  |
| 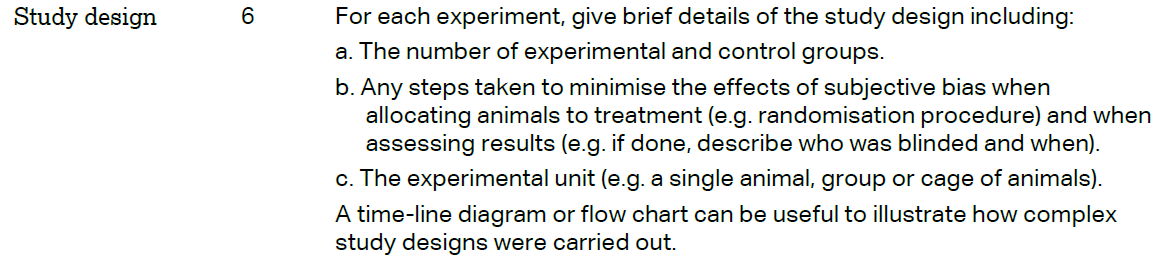 | | | Page 4-5, Methods, paragraph 3 |  |
| 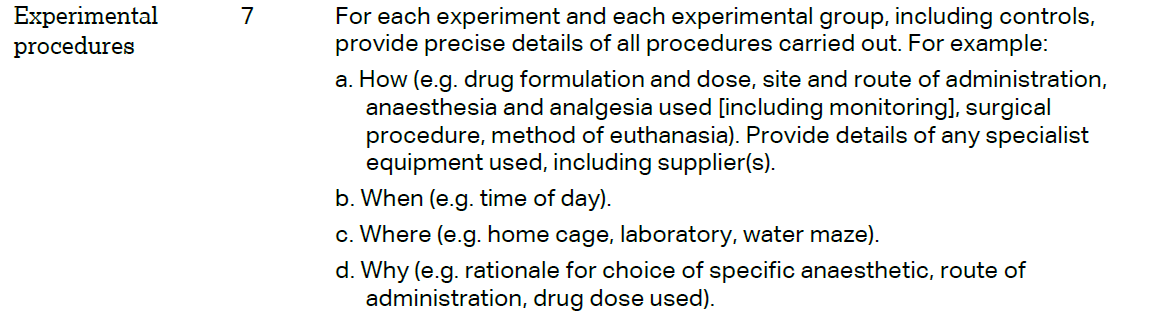 | | | Page 4-6 |  |
| 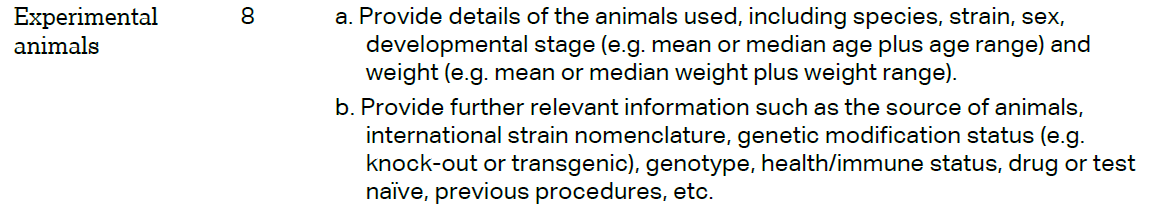 | | | Page 4-5, Methods, paragraph 3 |  |

The ARRIVE guidelines. Originally published in *PLoS Biology*, June 2010^1^

| 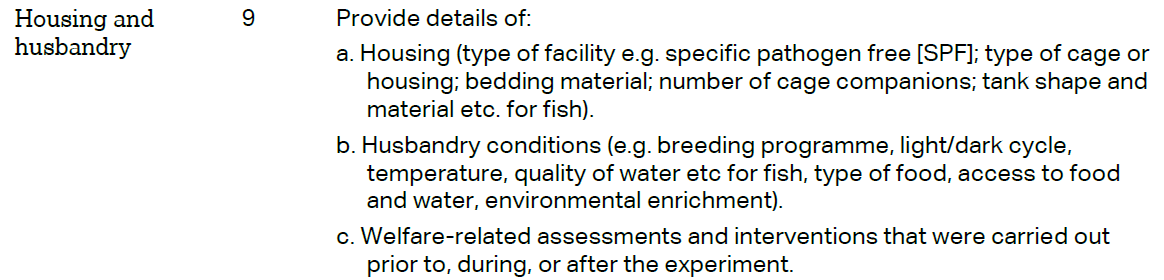 | Page 4-5, Methods, paragraph 3 | |
| --- | --- | --- |
| 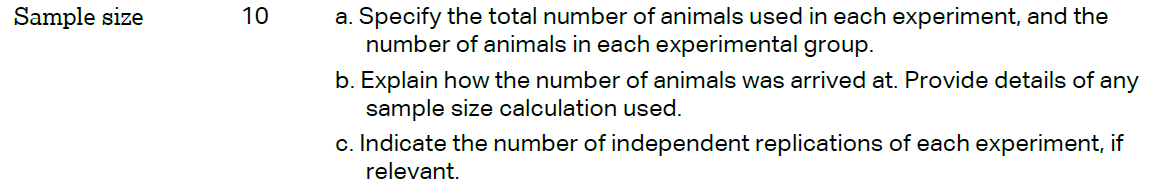 | Page 4-5, Methods, paragraph 3 | |
| 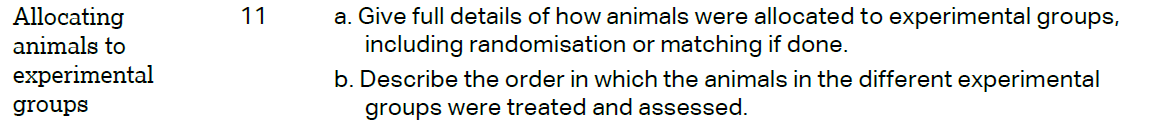 | Page 4-5, Methods, paragraph 3 | |
| 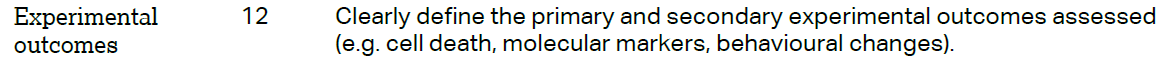 | Page 7-8 | |
| 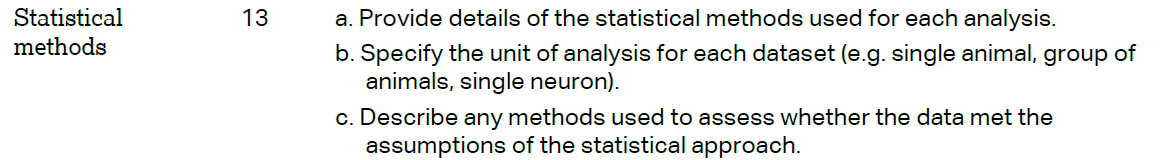 | N/A | |
| RESULTS |  | |
| 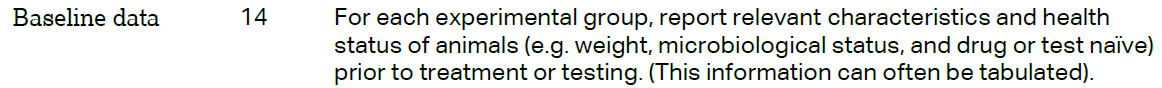 | Page 4-5  Page 7-8 | |
| 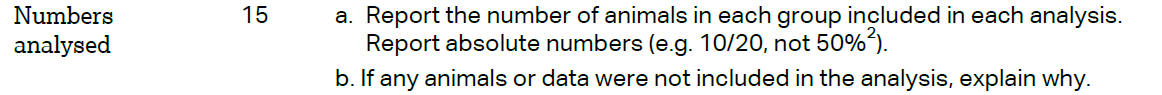 | Page 4-5  Page 7-8 | |
| 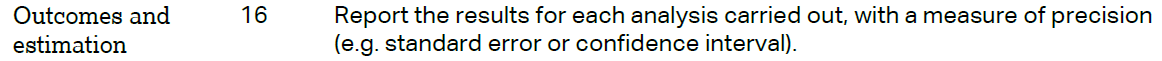 | Page 6-8 | |
| 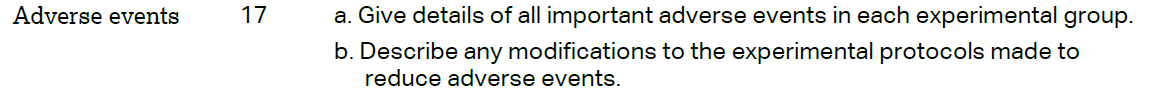 | N/A | |
| DISCUSSION |  | |
| 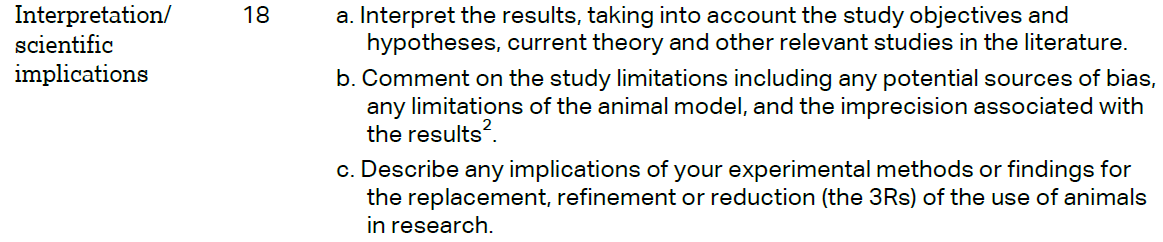 | Page 8-10  Discussion, | |
| 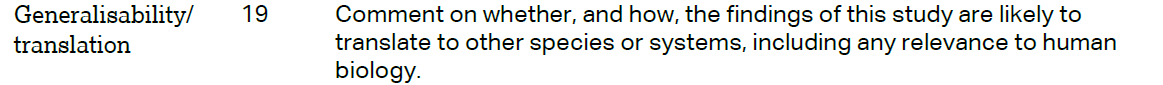 | Page 9-10  Conclusions | |
| 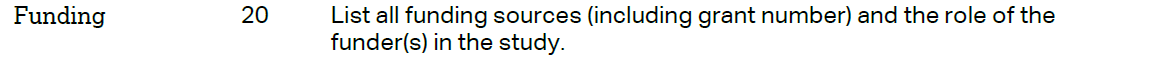 | | Page 10 |


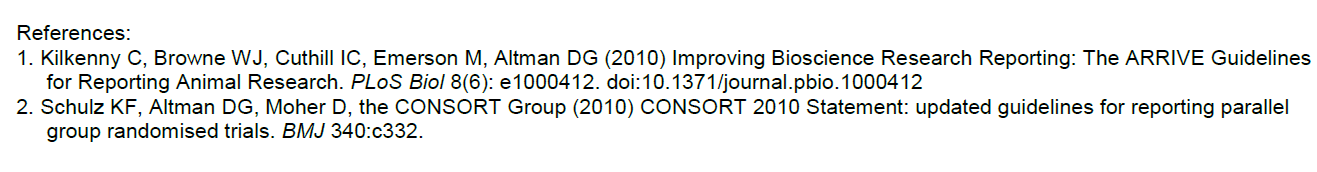

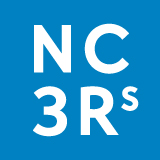

Supplement: Supplementary file 3 [file Table_2.DOCX]
